# Potential therapeutic targets of macrophages in inhibiting immune damage and fibrotic processes in musculoskeletal diseases

**DOI:** 10.3389/fimmu.2023.1219487

**Published:** 2023-07-20

**Authors:** Jianshu Zhu, Jiawei Fan, Yuanliang Xia, Hengyi Wang, Yuehong Li, Zijia Feng, Changfeng Fu

**Affiliations:** ^1^Department of Spine Surgery, The First Hospital of Jilin University, Changchun, China; ^2^Department of Gastroenterology, The First Hospital of Jilin University, Changchun, China

**Keywords:** macrophages, targeted therapy, immune damage, musculoskeletal diseases, inflammatory

## Abstract

Macrophages are a heterogeneous cell type with high plasticity, exhibiting unique activation characteristics that modulate the progression and resolution of diseases, serving as a key mediator in maintaining tissue homeostasis. Macrophages display a variety of activation states in response to stimuli in the local environment, with their subpopulations and biological functions being dependent on the local microenvironment. Resident tissue macrophages exhibit distinct transcriptional profiles and functions, all of which are essential for maintaining internal homeostasis. Dysfunctional macrophage subpopulations, or an imbalance in the M1/M2 subpopulation ratio, contribute to the pathogenesis of diseases. In skeletal muscle disorders, immune and inflammatory damage, as well as fibrosis induced by macrophages, are prominent pathological features. Therefore, targeting macrophages is of great significance for maintaining tissue homeostasis and treating skeletal muscle disorders. In this review, we discuss the receptor-ligand interactions regulating macrophages and identify potential targets for inhibiting collateral damage and fibrosis in skeletal muscle disorders. Furthermore, we explore strategies for modulating macrophages to maintain tissue homeostasis.

## 1 Introduction

The hallmark features of musculoskeletal disorders include persistent pain, tissue damage, and limited mobility (1, 2). Therefore, suppressing chronic inflammation and immune responses that cause collateral tissue damage in skeletal muscle disorders, as well as fibrosis resulting from the progression to the terminal stages of the disease, are important therapeutic targets for treating musculoskeletal disorders (3–5).

Macrophages comprise an incredibly diverse and heterogeneous group of cells (6). As their microenvironment constantly changes, macrophages are subject to various regulatory mechanisms that modulate their functional states to new set points in response to tissue alterations or environmental challenges (7, 8). Macrophages of different subpopulations and functional states play crucial roles in the pathogenesis and recovery of various musculoskeletal disorders (9). However, current treatments for musculoskeletal disorders do not always effectively restore the function of affected tissues, and existing therapeutic approaches lack specificity, necessitating the development of personalized, targeted treatment methods (10). The emergence of personalized treatment strategies in cancer therapy, such as immune checkpoint therapy (e.g., PD-1, PD-L1, and CTLA-4 inhibitors) and adoptive T cell therapy (e.g., CAR-T cells), provides inspiration for personalized treatment of musculoskeletal disorders (11, 12). The remarkable clinical success of immune checkpoint therapy and adoptive T cell therapy, as well as the improved understanding of immune cell biological functions, has greatly spurred interest in the field of targeted immunotherapy for musculoskeletal disorders (13–15).

Macrophages are central pathophysiological links in many disease states, such as the chronic inflammation and immune responses caused by their persistent activation, leading to the continuous progression of conditions like Osteoarthritis (OA), Rheumatoid arthritis (RA), and Systemic lupus erythematosus(SLE) (16, 17). Moreover, some studies have reported that macrophages in Systemic Sclerosis (SSc) can transform into myofibroblasts, playing an important role in the terminal stages of musculoskeletal disorders (18). In this review, we discuss recently discovered targets and mechanisms involving macrophage receptor-ligand interactions that activate or inhibit collateral tissue damage and fibrosis, as well as some ongoing clinical studies targeting macrophages for the treatment of musculoskeletal disorders. We aim to identify potential therapeutic targets for suppressing macrophage-induced collateral tissue damage and fibrosis.

## 2 Roles of macrophage subpopulations in immune-inflammatory injury and pathological fibrosis

**Figure 1:**
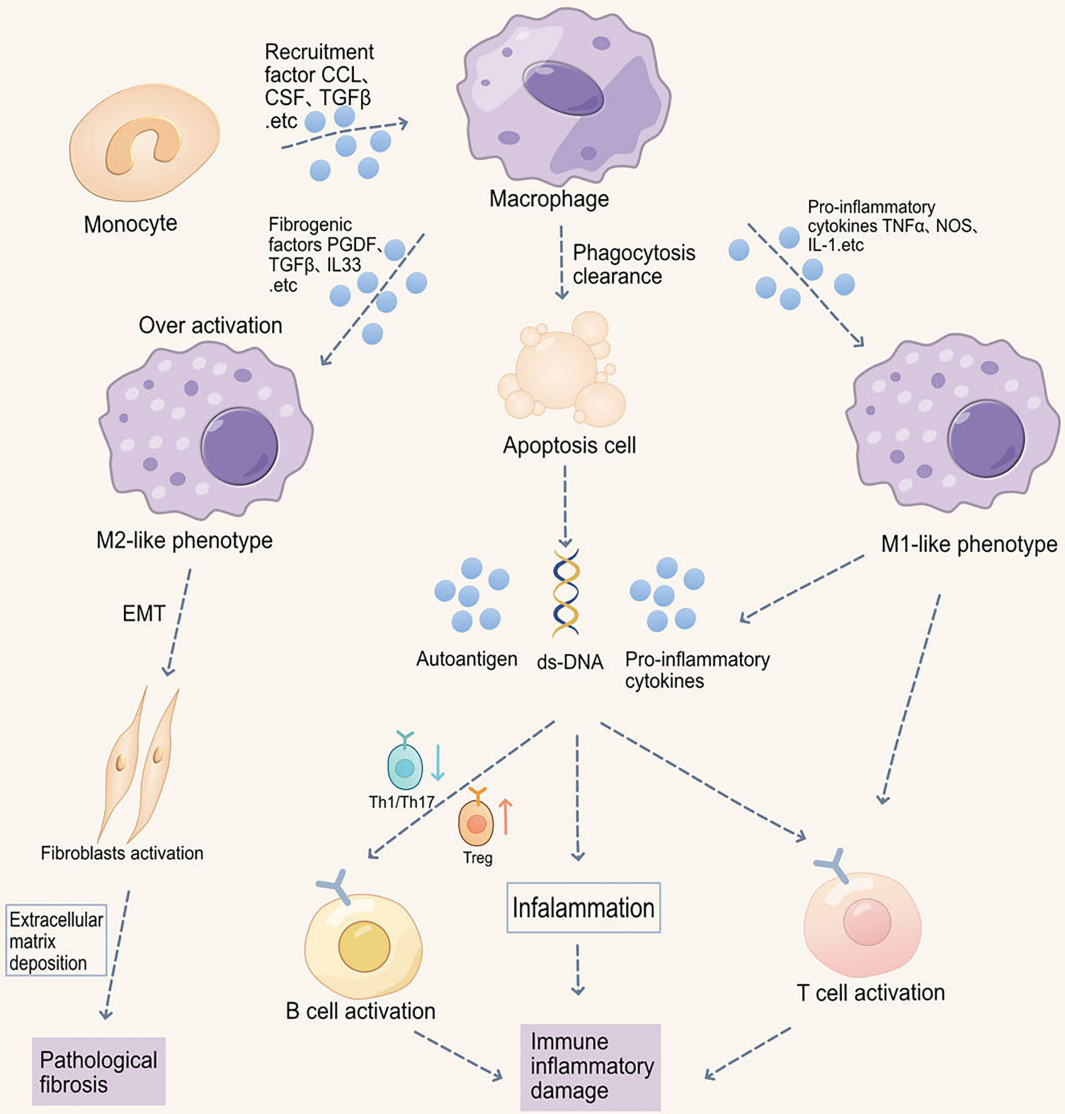
Macrophage-induced immune-inflammatory injury and pathological fibrosis in musculoskeletal disorders. Macrophage phagocytic function is impaired in musculoskeletal disorders, thereby inhibiting the clearance of apoptotic cells. Increased apoptotic cells promote the production of autoantigens and antibodies, exacerbating inflammation. Moreover, macrophages promote the migration and abnormal activation of T cells, including increased Th1/Th17 differentiation and downregulated Treg differentiation, ultimately leading to B cell abnormal activation. Imbalance in M1/M2 macrophage ratio also participates in autoimmunity. Abnormal M1 macrophage activation promotes the production of pro-inflammatory cytokines, such as IL-6, iNOS, TNF-α, and IL-1β, thereby promoting inflammation in target organs. Reduced M2 polarization impairs the production of anti-inflammatory cytokines and immune tolerance. Additionally, M2 macrophage receptor-ligand interactions can also cause epithelial-to-mesenchymal transition (EMT) and fibrosis in autoimmune diseases (e.g., SSc).

Macrophages play a crucial role in the initiation and resolution phases of inflammation, immune response, and pathological fibrosis in musculoskeletal system diseases (**Figure 1**) (19, 20). Monocyte-derived macrophages can differentiate into various macrophage phenotypes upon recruitment to tissues (21, 22). Changes in macrophage subpopulations and functions during inflammation and immune response are continuous, but corresponding surface markers are lacking (23). Therefore, in diseases of the musculoskeletal system, it is common to classify the course of the disease into different stages, namely, the initial stage of the acute phase, the inflammatory phase, the progressive phase of the subacute period, and the degenerative stage of the chronic period (24, 25). At different stages of the disease, musculoskeletal diseases exhibit distinct clinical and pathophysiological features (26). For instance, during the acute phase, which is often accompanied by severe inflammatory responses, pain, and functional impairment, symptoms usually manifest as pain, redness, increased heat, and restricted function. At this stage, macrophages promote inflammatory responses by secreting inflammatory mediators such as tumor necrosis factor-α (TNF-α), interleukin-1β (IL-1β), and interleukin-6 (IL-6). However, the progressive, subacute, and degenerative stages often accompany the ongoing progression of the disease and regressive changes, mainly manifesting as persistent chronic inflammation and tissue fibrosis (27, 28). Researchers typically classify macrophages into M1 pro-inflammatory and M2 anti-inflammatory macrophages based on their phenotypic and functional characteristics.

### 2.1 Abnormal function of M1 macrophages and their impact on immune inflammatory injury

Abnormal phagocytosis of M1 macrophages in autoimmune diseases may lead to imbalanced inflammation and immune responses (29). In various autoimmune diseases, such as rheumatoid arthritis (RA), systemic lupus erythematosus (SLE), and multiple sclerosis (MS), activation of M1 macrophages and sustained pro-inflammatory responses may exacerbate disease progression (30).

#### 2.1.1 Abnormal phagocytic function of M1 macrophages and immune inflammatory injury

Efficient phagocytosis by macrophages limits the release of intracellular PAMPs driving inflammation, thereby maintaining immune homeostasis (31, 32). In musculoskeletal diseases such as RA and SLE. The expression of inhibitory receptors, such as TIM-3, PD-1, CD32b, and CD200R, can suppress the activation and phagocytic function of macrophages by binding to corresponding ligands (33, 34). As a result, the phagocytic capacity of macrophages against pathogens and cell debris is inhibited [28;29]. Additionally, cytokines like IL-4, IL-10, and TGF-β, along with metabolic substances such as lipopolysaccharide (LPS) and high-density lipoprotein (HDL), can induce macrophage polarization towards M2 phenotype, and suppress macrophage activation and phagocytic function by binding to specific receptors (35–37). Furthermore, macrophage phagocytic capacity for pathogens and cellular debris is inhibited (38, 39). Impaired macrophage phagocytosis promotes the accumulation of uncleared apoptotic or necroptotic cells in autoimmune diseases (40). Increased apoptotic cells promote the production of autoantigens and antibodies, further exacerbating inflammation (41). M1 macrophages produce large amounts of pro-inflammatory cytokines, such as tumor necrosis factor-α (TNF-α), interleukin-1β (IL-1β), and interleukin-6 (IL-6), which intensify inflammation and cause tissue damage, playing a key role in chronic inflammation in RA, where their release of inflammatory cytokines leads to joint damage and disease worsening (42).

#### 2.1.2 Abnormal aggregation of M1 macrophages and disruption of immune tolerance

Abnormal accumulation of M1 macrophages may lead to the breakdown of immune tolerance, exacerbating the body’s attack on its own tissues (43, 44). The aberrant accumulation of M1 macrophages can regulate immune responses and tissue damage by affecting the activation and function of T cells (45, 46). In certain autoimmune musculoskeletal diseases, such as rheumatoid arthritis (RA), systemic lupus erythematosus (SLE), and multiple sclerosis (MS), the activation and accumulation of M1 macrophages lead to excessive Th1 and Th17 responses, thereby exacerbating tissue inflammation and damage (47–49). M1 macrophages produce large amounts of pro-inflammatory cytokines (such as TNF-α, IL-1β, and IL-6), causing massive release of PAMPs, thereby intensifying inflammation and tissue damage (50, 51). M1 macrophages can stimulate cell apoptosis, increase vascular permeability, and recruit more immune cells by producing inflammatory chemokines (such as CCL-2, CCL-3, and CXCL-10), creating a vicious cycle that further exacerbates the course of autoimmune diseases (29, 52, 53). Therefore, interventions targeting the recruitment and abnormal accumulation of M1 macrophages may have potential value in the treatment of musculoskeletal diseases such as RA, SLE, and MS (17, 54). Furthermore, in some fibrosis-related musculoskeletal diseases such as scleroderma (SSc), the abnormal polarization of M2 macrophages leads to an overactive TGF response, thereby promoting pathological tissue fibrosis (55).

### 2.2 Abnormal immunoregulatory function of macrophages and their impact on immune tolerance

Macrophages, vital components of the immune system, can activate T cells and regulate their function (56). However, an abnormal M1/M2 macrophage ratio may disrupt the immune balance, leading to overactivation or suppression of the immune system and potentially causing disruption of Immune Tolerance (57, 58).

#### 2.2.1 Abnormal antigen-presenting function of macrophages and disruption of immune tolerance

Macrophages are vital antigen-presenting cells (APCs) that can activate T cells by expressing major histocompatibility complex (MHC) molecules and presenting antigen fragments to T cell receptors (TCRs) (56). Macrophages can also regulate T cell polarization and function by expressing co-stimulatory molecules like CD80/CD86 and CD40, and by secreting cytokines such as IL-12 and IL-23 (56, 59). Macrophages can differentiate into different subtypes based on various stimulating factors and microenvironment conditions. M1 macrophages exhibit pro-inflammatory and immune-activating functions, promoting Th1 and Th17 cell differentiation and activation through the production of pro-inflammatory cytokines like IL-12 and IL-23 (60, 61). Conversely, M2 macrophages demonstrate anti-inflammatory and immunoregulatory functions, augmenting the function of regulatory B cells and regulatory T cells (Trg), and inhibiting Th1 and Th17 cell proliferation and differentiation through the production of anti-inflammatory cytokines like IL-10 and TGF-β (62). Moreover, studies have shown that the small protein RELMα, secreted by M2 macrophages, plays a crucial role and mechanism in IL-4-induced inflammatory responses. A deficiency in RELMα leads to a significant reduction in the number of FoxP3+ regulatory T cells. Macrophages expressing RELMα can directly promote the proliferation of regulatory T cells, thus limiting type 2 inflammatory responses (63).

#### 2.2.2 Abnormal immunoregulatory function of macrophages and disruption of immune tolerance

Abnormal innate immune response is an important cause of the collapse of autoimmune tolerance and macrophages are crucial components of the innate immune system (64). M2 macrophages possess strong anti-inflammatory and immune tolerance properties (6, 44). M2a, M2b, and M2c macrophages are anti-inflammatory intermediate macrophage subpopulations with immunoregulatory functions, mainly regulating inflammation and immune responses and participating in tissue repair and regeneration through the production of factors such as TGF-β, IL-6, and IL-10 (8, 53). M2a macrophages can enhance immune tolerance by secreting anti-inflammatory cytokines such as IL-10 and TGF-β, which induce T cells to polarize toward Th2 and inhibit T cell immune responses (65, 66). M2b macrophages are capable of secreting high levels of IL-10 and TGF-β and low levels of IL-12, which strongly regulate immunity and have anti-inflammatory effects. They inhibit the activation and differentiation of T cells such as Th1 and Th17 and NK cells, thereby reducing the risk of diseases such as autoimmune myositis (65, 67). Moreover, IgG4 can induce the transformation of M2a macrophages to an M2b-like phenotype by cross-linking the FcγRIIb receptor, thereby enhancing their ability to inhibit T cells (68). M2c macrophages can also induce immune tolerance by expressing inhibitory ligands such as PD-L1 to inhibit the activation and proliferation of T cells (68).Therefore, abnormal M1/M2 macrophage ratios may disrupt immune balance, leading to excessive activation or suppression of the immune system and, consequently, musculoskeletal diseases (17, 54). Macrophage migration and abnormal activation are related to T cell activation (53), including M1 macrophages producing pro-inflammatory cytokines like IL-12 and IL-23, which promote Th1 and Th17 cell differentiation (6). Conversely, M2 macrophages produce anti-inflammatory cytokines such as IL-10 and TGF-β, significantly enhancing the regulatory function of regulatory B cells, increasing Treg cell generation, and limiting T cell proliferation and differentiation into Th1 and Th17 cells (57, 58).

### 2.3 Abnormal function of M2 macrophages and their impact on tissue fibrosis

M2 macrophages are a macrophage subpopulation with tissue repair functions (44, 69, 70). If the factors causing tissue injury are not resolved, tissue inflammation induces macrophage polarization towards M2 type through IL-4 and IL-13-triggered signaling pathways such as STAT6 (8).

#### 2.3.1 Abnormal function of different M2 macrophage subsets and their impact on tissue fibrosis

M2a macrophages have the ability to inhibit inflammatory responses, promote tissue repair, and fibrosis. However, if persistently overactivated, they could lead to excessive tissue reconstruction and scar formation, resulting in pathological fibrosis.M2a macrophages express factors like TGF-β1, PDGF, and matrix metalloproteinases (71). These factors can promote the activation of myofibroblasts and the deposition of extracellular matrix, leading to fibrosis (71). In another study, it was found that M2a macrophages release exosomes containing factors such as TGF-β1, PDGF, and matrix metalloproteinases during pathological fibrosis (72). These factors can regulate the activation of myofibroblasts and deposition of extracellular matrix components, resulting in tissue fibrosis, promoting smooth muscle cell migration and adhesion, and causing vascular remodeling and pathological fibrosis (72–74). Moreover, research has found that by inhibiting histone deacetylase (HDAC) with Trichostatin A (TSA), the expression of pro-inflammatory and pro-fibrotic molecules in M2a macrophages can be reduced. This process also inhibits the activation of myofibroblasts, alleviating pathological fibrosis (75). It has been observed that M2b macrophages can mitigate tissue fibrosis by significantly inhibiting the proliferation, migration, and differentiation into myofibroblasts (MFs) of cardiac fibroblasts (CFs) through the suppression of the mitogen-activated protein kinase (MAPK) signaling pathway. Furthermore, they reduce the expression of fibrosis-related proteins such as collagen protein I (COL-1) and α-smooth muscle actin (α-SMA). This suggests that M2b macrophages may be utilized in protective treatments against pathological fibrosis (76, 77). M2c macrophages are generally considered to be macrophages with anti-inflammatory and tissue repair functions. However, if overactivated after an injury, M2c macrophages could promote excessive scar formation and fibrosis. They can lead to pathological fibrosis by enhancing the epithelial-mesenchymal transition of interstitial cells (78). Furthermore, by significantly reducing the M2c subgroup of M2 type macrophages through targeting M2 macrophages, pulmonary fibrosis can be effectively improved (79). Therefore, different subgroups of M2 type macrophages have dual immunomodulatory functions in musculoskeletal diseases. They can both inhibit autoimmune responses and promote tissue repair, but may also cause excessive fibrosis, atrophy, and disruption of immune tolerance.

## 3 Potential targets of macrophage receptor-ligand interactions

**Figure 2:**
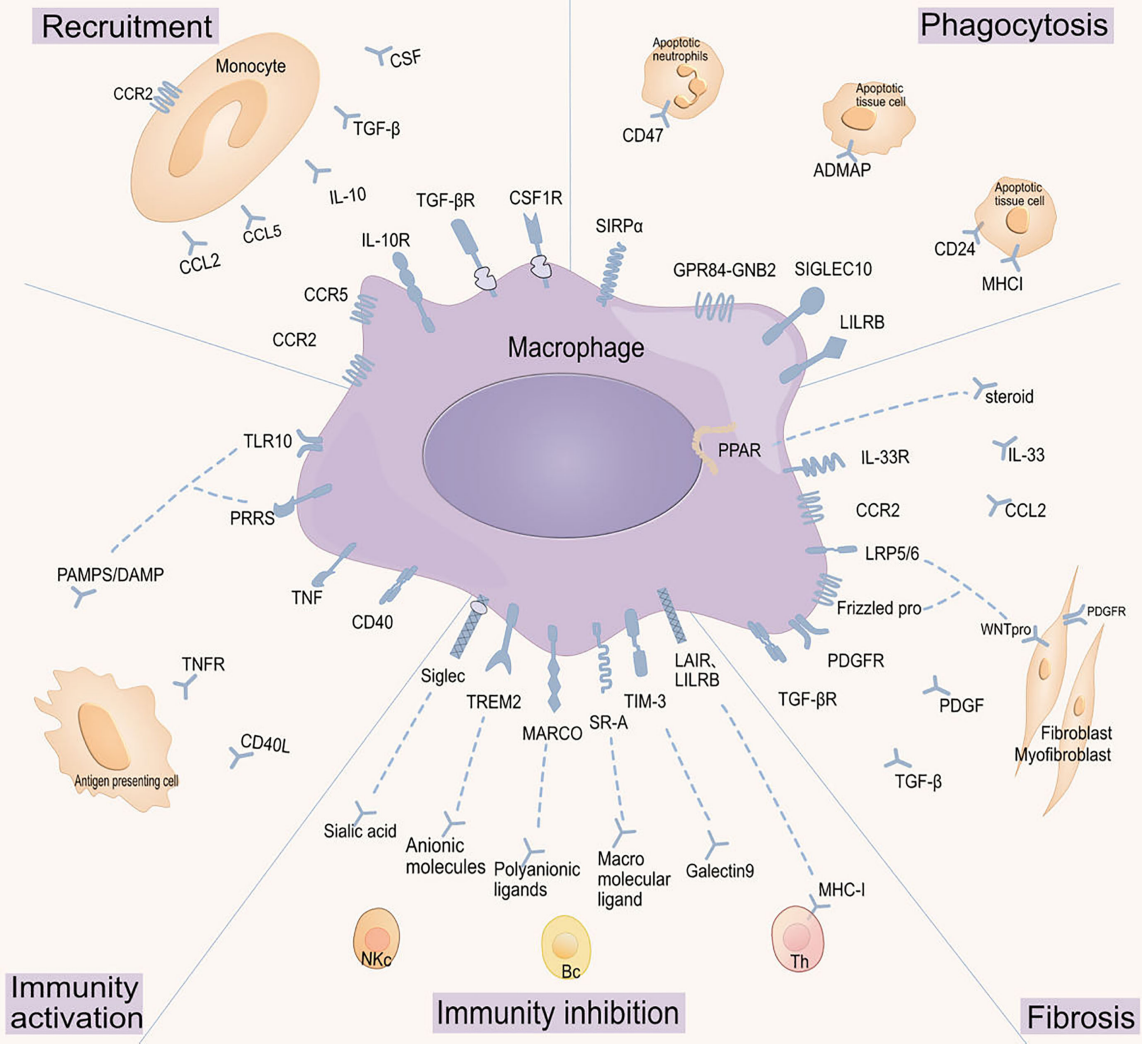
Targeting macrophage receptor-ligand interactions. Macrophage functions are regulated by various receptor-ligand interactions, which have been grouped according to their roles in macrophages: regulating macrophage cell recruitment, modulating phagocytic activity, activating macrophage immune functions, inhibiting macrophage immune functions, and regulating macrophage fibrotic activity. CCR2: C-C chemokine receptor type 2;CCL: Chemokine (C-C motif) ligand;IL-10R: Interleukin-10 receptor;IL-10: Interleukin-10;TGF-βR: Transforming growth factor-beta receptor;TGF-β: Transforming growth factor-beta;CSF1R: Colony-stimulating factor 1 receptor;CSF: Colony-stimulating factor;SIRP α: Signal regulatory protein α;CD47: Cluster of differentiation 47;GPR84-GNB2: G protein-coupled receptor 84 - G protein subunit beta 2;ADMAP: Adhesion and degranulation promoting adapter protein;SIGLEC10: Sialic acid-binding immunoglobulin-like lectin 10;CD24: Cluster of differentiation 24;LILRB: Leukocyte immunoglobulin-like receptor, subfamily B;MHCI: Major histocompatibility complex class I;PPAR: Peroxisome proliferator-activated receptor;IL-33R: Interleukin-33 receptor;IL-33: Interleukin-33;LRP5/6: Low-density lipoprotein receptor-related protein 5/6;WNTpro: Wnt protein;Frizzled pro: Frizzled protein;PDGFR: Platelet-derived growth factor receptor;PDGF: Platelet-derived growth factor;Sialec: Sialic acid-binding immunoglobulin-type lectin;TREM2: Triggering receptor expressed on myeloid cells 2;MARCO: Macrophage receptor with collagenous structure;SR-A: Scavenger receptor class A;TIM-3: T cell immunoglobulin and mucin-domain containing-3;LAIR: Leukocyte-associated immunoglobulin-like receptor;LILRB: Leukocyte immunoglobulin-like receptor, subfamily B;TNF: Tumor necrosis factor;PRRS: Porcine reproductive and respiratory syndrome;TLR10: Toll-like receptor 10;PAMPS/DAMP: Pathogen-associated molecular patterns/Damage-associated molecular patterns.

Macrophages are highly plastic and heterogeneous cells that utilize various surface receptors and secreted molecules to monitor and respond to environmental changes (51). Studies on receptor-ligand interactions between macrophages and other components of the immune microenvironment have identified key interactions that regulate macrophage function and abundance, maintaining tissue homeostasis and suppressing autoimmune inflammation and fibrosis (29, 80). Therefore, we have outlined a series of macrophage receptor-ligand interactions with therapeutic potential as potential treatment targets (**Figure 2**), along with their signaling pathways, biological benefits, and preclinical/clinical trials (**Table 1**).

**Table 1:** Macrophage receptor-ligand interaction signaling pathways, biological benefits, and preclinical/clinical trials.

Regulatory Types	Receptor-Ligand	Receptor Categories	Major Receptor Distribution	Signaling Pathways	Macrophage Polarization	Biological Effects	Preclinical/Clinical Studies	References
Recruitment Regulation	CCR2- CCL2	GPCR	monocyte、Macrophage	PI3K/Akt;MAPK: ERK1/2、JNK、p38;NF-κB	Induction of M1-type Macrophage Polarization	Promoting M1 Macrophage Formation and Migration; Inducing Inflammatory Response and Tissue Damage.	Inhibition of CCR2 suppresses recruitment of inflammatory monocytes (precursors of M1 Macrophages) and slows the progression of DMD	(81)
	CCR5- CCL5	GPCR	T cells、Macrophage、DC	PI3K/Akt;MAPK:ERK1/2、JNK、p38;NF-κB	Induction of M1-type Macrophage Polarization	Recruitment and Accumulation of Blood Monocytes	Oral CCR5 antagonist AZD5672 shows no clinical benefit in the treatment of RA	(82)
	CSF1R- CSF	Single-pass Transmembrane Receptor of RTK	Macrophage	PI3K/Akt、MAPK:ERK1/2、JNK和p38、JAK/STAT	Induction of M1-type Macrophage Polarization	M1 Pro-inflammatory Macrophage Infiltration as a Major Cause of Musculoskeletal Tissue Damage	Clinical efficacy observed with pexidartinib, a CSF1R inhibitor, in the treatment of tenosynovial giant cell tumor, with an ORR of 39%	(83)
	IL10R- IL10	Transmembrane Proteins of Type II Cytokine Receptor Family	Macrophage、T cells、NKC	JAK/STAT	Induction of M2-type Macrophage, Inhibition of M1-type Macrophage Activation	Anti-inflammatory and Immune-regulatory Functions; Maintaining Immune Homeostasis	Reduction in macrophage infiltration, inhibition of immune cell migration to inflammatory sites, and suppression of inflammation contribute to tissue damage attenuation	(84)
	TGFβR-TGFβ	RSK Family Receptors	Macrophage、T cells、B cells	Smad、MAPK、PI3K/Akt.etc	Induction of M2-type Macrophage, Inhibition of M1-type Macrophage Activation	Dual Role of TGFβ in Inhibiting or Suppressing Immune Damage	Importance of monocyte recruitment in the generation of M1 pro-inflammatory macrophages	(85)
	IL1a/βR- IL1a/β	Transmembrane Proteins of Type I Cytokine Receptor Family		MyD88-Dependent Pathway	Induction of M2-type Macrophage, Inhibition of M1-type Macrophage Activation	Strong Pro-inflammatory Effects; Potential Uncontrolled Inflammatory Response and Tissue Damage	Limited therapeutic effect as a monotherapy in treating knee osteoarthritis with synovitis	(86)
Phagocytic Checkpoints	SIRPα- CD47	Transmembrane Proteins of Immunoglobulin Superfamily	Macrophage、DC、neuron	SHP-1 and SHP-2 PTP Pathway *via* ITIM	Indirect Effects on Macrophage Polarization	Decreasing Macrophage Phagocytic Activity in the Immune Microenvironment	Inhibition of macrophage phagocytosis of erythrocytes; increased macrophage clearance of apoptotic cells by blocking ITIM pathway	(87)
	SIGLEC10- CD24-	CD24:Glycoprotein SIGLEC10:Transmembrane Glycoprotein of the Immunoglobulin Superfamily	CD24:广泛分布 SIGLEC10:Macrophage、DC、B cells	SHP-1 and SHP-2 PTP Pathway *via* ITIM	Indirect Effects on Macrophage Polarization	Anti-phagocytic, Inhibition of Immune Cell Activation; Prevention of Immune Cell Overactivation	\	(88)
	GPR84-APMAP	GPCR	Macrophage、Neutrophils	\	Induction of M1-type Macrophage Polarization	Decreasing Macrophage Phagocytic Activity in the Immune Microenvironment	Loss of APMAP expression enhances phagocytic function, promotes apoptotic cell engulfment, suppresses autoimmune reactions triggered by antigen presentation, and restricts immune-inflammatory damage	(89)
	LILRB1-MHC I	Inhibitory Receptors of the Leukocyte Immunoglobulin-like Receptor Family	Macrophage、monocyte、DC	SHP-1 and SHP-2 PTP Pathway *via* ITIM	Indirect Effects on Macrophage Polarization	Inhibiting Macrophage Activation, Reducing Activity in the Immune Microenvironment, Preventing Immune Cell Overactivation, Maintaining Tissue Homeostasis	\	(90)
Immune Stimulation	PRRs-PAMP/DAMP	TLRs、CLRs、NLRs、RLRs	Macrophage、DC	TLRs, CLRs: NF-κB and IRF Signaling Pathways; NLRs and RLRs : Caspase-1 Pathway	Induction of M1-type Macrophage Polarization	Induction of Inflammatory Cytokines and Type I Interferon Production	Overactivation of PRRs may lead to immune damage	(91)
	TLR4- LPS/DAMP		Macrophage、DC、Neutrophils	MyD88-Dependent Pathway: NF-κB and MAPK Pathways; TRIFDependent Pathway: IRF3 and NF-κB Pathways	Induction of M1-type Macrophage Polarization	Strong Inflammatory Response and Antipathogenic Ability; Persistent Activation of TLR4 Signaling May Lead to Uncontrolled Inflammatory Response	Humanized anti-TLR4 monoclonal antibody Paridiprubart shows potential for rheumatoid arthritis research by promoting macrophage apoptosis and inhibiting Th1 response	(92)
	TNFR- TNF		TNFR1 Widely distributed、TNFR2:Macrophag、endothelial cells.	TNFR1 Activation of Downstream NF-κB, MAPK, PI3K/Akt, and JNK Signaling Pathways	Induction of M1-type Macrophage Polarization	Inducing Inflammatory Response, Causing Immune Damage	Anti-TNF drugs reduce concentrations of matrix metalloproteinases MMP-1 and MMP-3, cartilage and synovial proliferation, acute phase inflammation markers CRP and ESR, pro-inflammatory cytokine production in monocyte-derived macrophages, and increase phagocytic function	(93)
	CD40- CD40L	CD40L/CD40Transmembrane Glycoprotein, Member of the Tumor Necrosis Factor (TNF) Family	CD40L、CD40:T cells、B cells、Macrophage、DC.	NF-κB、JNK、p38 MAPK	Induction of M1-type Macrophage Polarization	Promoting Antigen Presentation Ability of Antigen-Presenting Cells, Affecting T-cell Activation and Function, Overactivation May Lead to Autoimmune Response Dysregulation and Damage to Normal Tissues	CD40L monoclonal antibody Toralizumab blocks CD40 signaling, providing protection in multiple sclerosis and potential treatment for systemic lupus erythematosus	(94)
Immune Suppression	TREM2- Anionic molecules	Transmembrane Immunoglobulin, Member of the Triggering Receptor Family (TREM Family)	Macrophage、Microglial cells	Activation of SYK, PI3K/AKT, and ERK Signaling Pathways *via* TAM	Induction of M2-type Macrophage Polarization	Inhibiting Inflammatory Response, Suppressing Pro-inflammatory Cytokine Production in Macrophages, Increasing Arginase 1 Expression, and Expressing IFNγ to Inhibit T-cell Function, Promoting Neuroprotection and Regulating Cell Survival	In neuromuscular degenerative diseases, TREM2 plays a key role in modulating microglial function and neuroinflammation; TREM2 mutations or functional defects are closely related to the onset and progression of neurodegenerative diseases	(95)
	MARCO- Polyanionic ligands	Transmembrane Glycoprotein, Member of the Scavenger Receptor Family	Macrophage(Especially on the surface of plasma cell like macroscopic).		Induction of M1-type Macrophage Polarization	Regulating Macrophages, Leading to Inhibition of Natural Killer Cell and T-cell Activation, and Increased Infiltration of Regulatory T-cells (Treg Cells), Exhibiting Immunosuppressive Function in the Immune Microenvironment	MARCO+ monocytes are potent effector cells for skin and lung fibrosis in SSc, with their presence correlating with disease onset and progression	(96)
	SR-A— macromolecular ligand	Transmembrane Glycoprotein, Member of the Macrophage Scavenger Receptor Family	Macrophage	MAPK、NF-κB 、JAK/STAT	Induction of M1-type Macrophage Polarization	Participating in the Clearance of Endogenous Waste, Alleviating Inflammatory Response, and Inhibiting Immune Damage through Suppression of T-cell Function	SR-A plays a key role in chronic inflammatory diseases; SR-A neutralizing antibody is a potential candidate drug for improving rheumatoid arthritis-associated osteolysis	(97)
	TIM3- galectin 9	Transmembrane Immunoglobulin, Member of the TIM Family	Macrophage、TC、DC、NKC、BC,		Induction of M2-type Macrophage Polarization	Inhibiting the Activation and Effector Functions of T-cells and Other Immune Cells, Limiting the Development of Chronic Inflammation and Immune Damage	TIM3 plays a crucial role in bone marrow cell-mediated inflammatory responses	(98)
	Siglec- sialic acid	Transmembrane Glycoprotein, Member of the Siglec Family	Macrophage、NKC、Monocyte	ITIM-Mediated Activation of Immune Cell Inhibition Signaling Pathways	\	Inhibiting Inflammatory Response and Cytokine Production, Reducing Neutrophil and Macrophage Migration and Chemotaxis, Regulating T-cell and Antigen-Presenting Cell Interactions, Suppressing Immune Damage	Siglec-15 exhibits multiple functions in osteoclast development, bone resorption, and T cell immunity	(99)
	LAIR1、LILRB2、LILRB4- MHC I	Transmembrane Glycoprotein, Member of the Immunoglobulin Superfamily	Monocyte、Macrophage、DC、B celsl	ITIM-Mediated Activation of Immune Cell Inhibition Signaling Pathways	\	Involvement in Treg Cell Recruitment, Regulating Macrophage Function in the Immune Microenvironment, Maintaining Immune System Homeostasis, Alleviating Inflammatory Response, Preventing Overactivation of the Immune System	Overexpression of LAIR-1 in macrophages within synovial tissue of RA patients	(100)
Fibrosis Regulation	PDGFR- PDGF	Tyrosine Kinase Receptor	Fibroblasts、smooth muscle cell、endothelial cells、Macrophage	Ras/MAPK、PI3K/Akt、PLCγ/PKC、STAT Pathways	Induction of M2-type Macrophage Polarization	Tissue Repair, Angiogenesis, Inflammatory Response, Tumor Growth, Inducing Fibrosis	Under fibrotic conditions, transforming growth factor β (TGF-β) is enhanced, while platelet-derived growth factor (PDGF) signaling is upregulated in regenerative conditions	(101)
	IL33R**-**IL33	IL-1 Family Members	Multiple cell types	Activation of MyD88-Dependent Signaling Pathways, Activating NF-κB and MAPK Pathways	Induction of M2-type Macrophage Polarization	Recruiting and Regulating Inflammatory Cell Function, Producing Pro-fibrotic Cytokines IL-13 and TGF-β1, Leading to Pathological Fibrosis Development	Polarized M2 macrophages produce IL-13 and TGF-β1, enhancing profibrotic cytokine production and promoting fibrosis onset and progression	(102)
	PPAR- steroid	Nuclear Receptor	Expressed in multiple tissues and cell types	PPARs Binding with Nuclear Receptor RXR, Regulating Target Gene Expression through PPRE	Induction of M2-type Macrophage Polarization	Directly Regulating Macrophage Activation without Affecting Infiltration, Effectively Counteracting Inflammation and Fibrosis Disease Progression	PPAR agonists effectively counteract inflammation and disease progression, improving tissue inflammation and fibrosis	(103)
	CCR2-CCL2	GPCR	Monocyte、Macrophage、T cell subpopulations (Th1, Th17)	PI3K/Akt、MAPK、PLC/PKC、Rho GTPase Pathways	Inducing macrophages to polarize toward M2 phenotype to some extent	Inducing Macrophage Accumulation in Damaged Tissue; Macrophage Infiltration Exacerbating Inflammatory Response, Leading to Fibrosis Development	In some fibrotic diseases, CCL2/CCR2 signaling has been implicated in the pathological process, and its inhibition has therapeutic potential	(104)
	TGFβR-TGFβ	Membrane-bound Receptor	Macrophage、T cells和B cells、epithelial cells、Fibroblasts	Smad、MAPK、PI3K/Akt、Rho GTPase Pathways	\	Activating Macrophages and Inducing Fibroblasts to Transition to a Pro-fibrotic Activated State	Consistent antifibrotic activity of TGFβ-blocking agent pirfenidone in various animal models	(105)
	Frizzled protein、LRP5/6- Wnt protein	Frizzled protein:GPCR、LRP5/6:Low-Density Lipoprotein Receptor-Related Protein	Widely distributed	Canonical、non-canonical Pathways、Wnt Pathways	Induction of M2-type Macrophage Polarization	Wnt pathway activation closely associated with EMT, activation of fibroblasts, and extracellular matrix deposition	Drugs and small molecule inhibitors targeting the Wnt signaling pathway have shown therapeutic effects in animal models of fibrotic diseases; inhibition of Wnt signaling has antifibrotic therapeutic potential	(106)

GPCR, G protein-coupled receptor; RXR: Retinoid X receptor; RTK, Tyrosine kinase-like receptor; canonical:β-catenin-dependent non-canonical:β- Chain protein independence; NLRs, Nucleic acid-sensing receptors; EMT, epithelial-mesenchymal transition; CLRs, C-type lectin-like receptors; DMD, Duchenne Muscular Dystrophy; SR A, Scavenger Receptor A; RSK, serine/threonine kinase receptor; RLRs, RIG-I-like receptors; TLRs, Toll-like receptors; ITIM, Immunoreceptor tyrosine-based inhibitory motif.

### 3.1 Recruitment and aggregation: regulation of macrophage cell abundance

Recruitment and accumulation of macrophages are related to the prognosis and treatment effects of musculoskeletal disorders. The accumulation of macrophages in blood vessels and interstitial tissue is a significant feature of acute and chronic inflammatory musculoskeletal disorders (9). Macrophages that accumulate in damaged musculoskeletal tissues are primarily derived from bone marrow monocytes (107), and their local proliferation is a characteristic of inflammatory damage (108).

Various chemokines (such as CCL-2, CCL-5, CXCL-9, CXCL-10) and cytokines (such as IL-4, IL-10, IL-13, IL-1, TGF-β) play roles in the recruitment and polarization of macrophages (44, 109–111). Chemokine axes like CCL-2-CXCR-2, CCL-5-CCR-5, and CSF-CSF-1R, and cytokine interactions like IL-10-IL-10R, TGF-β-TGF-βR play key roles in the recruitment and function of suppressive macrophages (110, 112–114). These studies demonstrate the importance of monocyte recruitment for the generation of M1 pro-inflammatory macrophages (31–33) and establish that infiltration of M1 pro-inflammatory macrophages is a major cause of tissue damage in musculoskeletal disorders (115–117) and establish that infiltration of M1 pro-inflammatory macrophages is a major cause of tissue damage in musculoskeletal disorders (34–36). In preclinical studies, targeting these pathways has led to a significant reduction in the recruitment and accumulation of blood monocytes (85, 118, 119). In preclinical studies, targeting these pathways has led to a significant reduction in the recruitment and accumulation of blood monocytes (120, 121). The reduction of macrophage infiltration, inhibition of immune cell arrival at the site of inflammation, and suppression of inflammatory responses can mitigate tissue damage (51, 81, 84). Although there are clear reasons to target CCR-5, clinical studies on patients with active rheumatoid arthritis (RA) have shown that oral CCR-5 antagonist AZD5672 provides no clinical benefits, suggesting that the use of CCR-5 antagonists alone is unlikely to be a viable treatment strategy for RA (82).

Research shows that M1 macrophages play a key role in the development of Duchenne muscular dystrophy (DMD). Inhibiting CCR-2 suppresses the recruitment of inflammatory monocytes (precursors of M1 macrophages) and slows the progression of DMD (83). In addition, patients with tenosynovial giant cell tumors caused by genetic translocation-induced CSF1 overexpression have shown clinical efficacy when treated with the CSF-1R inhibitor pexidartinib, with an ORR of 39% (86). However, the use of IL-1a/β inhibitors as monotherapy for treating knee osteoarthritis with synovitis has limited therapeutic effects (122, 123). The suboptimal therapeutic effect of macrophage chemokine blockade might be due to the heterogeneity of macrophage populations in the immune microenvironment and the differential effects of these targeting strategies (51, 124).

Furthermore, the activation of the complement cascade can drive the recruitment of monocytes to damaged tissues, resulting in the deposition of complement component C3b and the local release of effective chemotactic molecules C3a and C5a (125). The complement system plays a role in promoting macrophage recruitment in chronic inflammatory demyelinating polyneuropathy (CIDP), generating a pro-inflammatory environment and mediating demyelination (126–128).

### 3.2 Phagocytic Checkpoint Receptor-Ligand Interactions

Macrophages’ phagocytosis and clearance of apoptotic cells are essential for suppressing autoimmune diseases (129). In musculoskeletal disorders such as rheumatoid arthritis (RA) and systemic lupus erythematosus (SLE), the expression of phagocytosis-related receptors on macrophages (e.g., Fc receptors and complement receptors) may change, thereby affecting their phagocytic capacity (130, 131).

Anti-phagocytic signals in tissue cells, such as CD47 and CD24, interact with signal regulatory protein-alpha (SIRP-α) and sialic acid-binding immunoglobulin-like lectin 10 (SIGLEC10), both of which are highly expressed on monocytes and macrophages, leading to the inhibition of macrophages’ phagocytic function towards apoptotic cells (88, 132). For example, CD47 acts as a self-marker on red blood cells, interacting with SIRPα on macrophages to inhibit the phagocytosis of red blood cells (87).

Although immune checkpoint therapy is primarily used for cancer treatment, its application in autoimmune diseases has been explored in recent years (133–135). Therefore, CD47-SIRPα axis-targeted therapies are currently being investigated in various clinical trials, with blockade of this pathway enhancing macrophages’ ability to phagocytose and clear apoptotic cells (136, 137). Studies using mouse models of autoimmune diseases have found that CD47-SIRPα and CD24-SIGLEC10 polymorphisms affect macrophages’ phagocytosis of apoptotic cells, suggesting that modulating the CD24-SIGLEC10 axis may have potential value in various musculoskeletal disorders (87, 88). Although there are not yet many clinical trial reports on CD24-SIGLEC10-targeted therapies for autoimmune diseases, existing research provides a foundation for further exploration in this field (88, 138). In the future, more studies and clinical trials may focus on the application of CD47-SIRPα and CD24-SIGLEC10 axes in autoimmune diseases.

Despite the promise of CD47 and SIRPα as clinical targets, the widespread expression of CD47 has led to different off-target effects and responses, posing challenges for the development of clinical treatments targeting these molecules (139, 140). Recent research has identified additional phagocytic checkpoints that alter macrophages’ phagocytic function. One such checkpoint is the G-protein coupled receptor GPR84 and its signaling partner GNB2, which interact with the anti-phagocytic factor APMAP expressed on tissue cells, resulting in enhanced phagocytosis in APMAP-deficient cells (89, 141, 142). When APMAP is knocked out, the level of CD47 on the cell surface decreases, making it easier for macrophages to recognize and engulf them (89, 143). Adipocyte plasma membrane-associated protein (APMAP) is ubiquitously expressed in all cell lines and various types of musculoskeletal disorders (89), APMAP deficiency synergizes with CD47-blocking monoclonal antibodies to enhance phagocytic function, promotes the engulfment of apoptotic cells, suppress antigen presentation-induced autoimmune responses, limit immune-inflammatory damage, and contribute to tissue homeostasis maintenance (144–146). Another phagocytic checkpoint is the LILRB1 on the surface of macrophages, which is an inhibitory immunoglobulin-like receptor. It can bind to MHC class I molecules expressed on tissue cells, reducing macrophages’ phagocytic activity in the immune microenvironment (90, 147, 148). Thus, these newly discovered receptor-ligand interactions may become important therapeutic targets in the future.

### 3.3 Immunoregulation

Similar to the modulation of T cells through the activation and inhibition of checkpoint receptors, the immunostimulatory and immunosuppressive roles of macrophages are also regulated by various modulatory molecules (30, 149, 150). Macrophages express multiple receptors that interact with various ligands on different cells in the immune microenvironment, which have been shown to reduce the extent and duration of inflammatory responses and, in some cases, contribute to the resolution of fibrosis (16, 84). In this section, we discuss the newly discovered macrophage activation and inhibitory receptors that may play important roles in limiting immune damage in musculoskeletal disorders.

#### 3.3.1 Immune stimulatory receptor-ligand interactions

The activation of M1 macrophages is mainly stimulated by pathogen-associated molecular patterns (PAMPs) and cytokines produced by Th1 cells, such as interferon-γ (IFN-γ). Type I and II interferon responses mediate immune damage responses through intrinsic cellular cytotoxicity and immune activation (70, 151, 152). Pattern recognition receptors (PRRs) are primarily expressed on antigen-presenting cells (APCs), including macrophages and dendritic cells (153, 154). The interaction between PRRs and PAMPs/DAMPs activates macrophages and dendritic cells, leading to the expression of pro-inflammatory cytokines and other immunoregulatory molecules and enhancing their immunostimulatory effects (154–156).

Co-stimulatory molecules of the tumor necrosis factor (TNF) receptor superfamily play an essential role in initiating T cell responses in dendritic cells (DCs) (157–159). Pro-inflammatory cytokines can be induced in macrophages by stimulating Toll-like receptor 7 (TLR7) and TLR9 with CL097 or the interferon gene stimulator (STING) (160, 161). Preclinical studies have shown that activation of PRRs can cause immune damage (91, 162), suggesting that these PRRs are essential targets for immunotherapy (91, 155).TLR agonists are critical targets for immunotherapy because they bridge the gap between the innate and adaptive immune systems (92, 163). Currently, ligands for different members of the TLR family are being studied as potential therapeutic agents, both as monotherapies and in combination with other immunotherapies. Paridiprubart (NI-0101) is a humanized anti-TLR4 monoclonal antibody (93). Paridiprubart has potential in rheumatoid arthritis research by promoting macrophage apoptosis and inhibiting Th1 responses to reduce macrophage accumulation (93). Adalimumab, an anti-TNF biologic antirheumatic drug, has been shown *in vitro* to block the interaction of TNF with p55 and p75 cell surface TNF receptors, reduce the concentrations of matrix metalloproteinase MMP-1 and MMP-3, reduce cartilage and synovial proliferation, and decrease the concentrations of acute-phase inflammatory reactants (CRP and ESR), reduce the production of pro-inflammatory cytokines by monocyte-derived macrophages and increase phagocytosis (164–166), all of which alleviate the inflammatory response and limit immune damage.

MHCII and co-stimulatory molecules expressed on macrophages (such as CD40, CD80, and CD86) promote T cell activation (92, 167). CD40L-CD40 binding can activate dendritic cells (DCs), and activated DCs promote T cell differentiation and trigger effective CTL responses by enhancing the expression of B7 molecules and the secretion of cytokines such as interleukin-12 (IL-12) (168, 169). Various STING and CD40 agonists are also being tested in clinical trials, either as monotherapies or combination therapies. Bleselumab (ASKP 1240) is a human anti-CD40 monoclonal antibody (mAb) that binds human CD40 with high affinity and inhibits immune responses by blocking the interaction between CD40 and its ligand CD40L (170, 171). Ruplizumab (BG 9588) is a humanized monoclonal anti-CD40L (TNF Receptor) IgG1 antibody with potential for use in systemic lupus erythematosus research (94). Toralizumab (IDEC-131) is a humanized monoclonal antibody (mAb) targeting CD40L (CD154) that specifically binds to human CD40L on T cells, thereby blocking CD40 signaling. Toralizumab, as an immunosuppressive agent, has been shown to be safe and effective in multiple sclerosis research (172), and also has potential for use in active systemic lupus erythematosus (SLE) research. Therefore, it is necessary to better understand the mechanistic basis of their modes of action to optimize their immunotherapeutic efficacy. When developing therapeutic strategies targeting these receptors, factors such as the duration of receptor-ligand interactions, the type of exposed cells, and the nature of the inflammatory environment in the immune microenvironment should be carefully considered.

#### 3.3.2 Immune inhibitory receptor-ligand interactions

Inhibitory receptors on macrophages suppress the activation of pro-inflammatory myeloid cells, skewing their function towards an immunosuppressive phenotype. M2 macrophages primarily create an anti-inflammatory environment in the immune milieu by producing anti-inflammatory cytokines, such as IL-10 and TGF-β, which help maintain tissue homeostasis (173, 174). Their activation is mainly regulated by cytokines produced by Th2 cells, such as IL-4 and IL-13 (51, 175). In this section, we highlight several novel inhibitory receptors with promising therapeutic potential.

PRR-dependent immune injury responses are mainly driven by interferons. However, other studies have shown that interferons can also exert immunosuppressive effects (176–178). Chronic interferon signaling can increase the expression of immune checkpoint ligands such as PDL1 and PDL2 and immunosuppressive molecules, thereby limiting immune injury (179, 180). Scavenger receptors are widely expressed on immune cells, particularly macrophages, and exert immunosuppressive effects through phagocytosis and regulation of inflammatory responses (181). In the immune microenvironment, TREM2-mediated signaling pathways in macrophages suppress the production of pro-inflammatory cytokines, increase the expression of arginase-1, and express IFNγ to inhibit T cell function (182–184). In neuromuscular system neurodegenerative diseases, the TREM2 receptor on macrophages plays a crucial role in regulating microglial function and neuroinflammation. Mutations or functional defects in TREM2 are closely related to the onset and progression of neurodegenerative diseases (95, 185). MARCO interacts with multiple anionic ligands, including nucleic acids, anionic proteins, and lipids, modulating macrophages, inhibiting the activation of natural killer cells and T cells, and increasing the infiltration of regulatory T cells (Tregs), indicating its suppressive function in the immune microenvironment (96, 186). Moreover, research shows that the MARCO+ macrophage subpopulation is associated with driving the onset and progression of diffuse cutaneous systemic sclerosis (SSc), and MARCO+ monocyte-derived macrophages are potent effector cells causing tissue fibrosis (96, 187). SR-A (Scavenger Receptor-A) participates in the clearance of endogenous waste and alleviates inflammatory responses by inhibiting signaling pathways such as NF-κB and MAPK, and suppresses immune injury by inhibiting T cell function. Thus, targeting these scavenger receptors may be a promising approach (97, 188, 189). Blocking SR-A with an anti-SR-A neutralizing antibody may offer a hopeful treatment strategy for bone destruction in RA (97). In addition, some receptors of the immunoglobulin family have been found to promote inhibitory functions. For example, TIM-3 (T cell immunoglobulin and mucin domain-containing protein 3) is mainly expressed on T cells (CD4^+^ Th1, CD8^+^ subsets), macrophages, and dendritic cells (190). When TIM-3 binds to its ligand galectin-9, it inhibits the activation and effector functions of T cells and other immune cells, thereby exerting immunosuppressive effects (191, 192). In addition to its inhibitory effects on Th1 cells, recent compelling experiments have emphasized the indispensable role of TIM-3 in bone marrow cell-mediated inflammatory responses (98). The Siglec family (Sialic Acid-Binding Immunoglobulin-like Lectins), such as Siglec-9, upon interaction with ligands, suppresses inflammatory responses and cytokine production, reduces the migration and chemotaxis of neutrophils and macrophages, and regulates the interaction between T cells and dendritic cells, further inhibiting immune responses (193–195). Siglec-15 is an immune receptor that plays multiple roles in osteoclast development, bone resorption, and macrophage-mediated T cell immune responses, serving as a potential target for the treatment of osteoporosis (99, 196). Additionally, LILRB2 and LILRB4, along with the related receptor Leukocyte-Associated Immunoglobulin-like Receptor 1 (LAIR1), are involved in the recruitment of Treg cells and regulation of macrophage function in the immune microenvironment (197, 198). LAIR-1 is highly expressed in CD14(+) mononuclear cells and local CD68(+) macrophages in the synovial tissue of RA patients. Upon TNF-α stimulation, LAIR-4 expression in helper T cells (Th)1 and Th1 CD2(+) T cells from healthy donors is reduced. These results suggest that LAIR-1 exerts distinct functions on T cells and mononuclear cells/macrophages and indicates that LAIR-1 may be a novel therapeutic target for RA (100).

The aforementioned preclinical studies demonstrate that targeting these receptors can reverse the immune damage effects in various musculoskeletal diseases and restore tissue homeostasis, suggesting the potential of these receptors as macrophage-specific targets for monotherapy or in combination with other immunotherapeutic drugs. Consequently, various monoclonal antibodies targeting biomolecules produced by macrophages can be used as therapeutic options for RA. Notably, both inhibitory and activating receptors are widely expressed in various immune and non-immune cell subpopulations in the immune microenvironment.

### 3.4 Fibrosis regulation

Musculoskeletal diseases are characterized by limited activity due to fibrosis, such as systemic sclerosis, which is a musculoskeletal disease characterized by fibrosis of various tissues (199). Multiple studies have linked the fibrotic features of M2 macrophages to the pathogenesis of this disease. Myofibroblasts are generated from various sources, including the epithelial/endothelial-to-mesenchymal (EMT/EndMT) transition process, as well as circulating fibrocyte-like cells derived from bone marrow stem cells (200). Activated M2 macrophages are particularly abundant in the blood and skin of patients with systemic sclerosis and have been shown to be potential major sources of fibrosis. Tissue fibrosis is an aberrant pathological process involving excessive extracellular matrix (ECM) deposition, resulting in impaired tissue structure and function (201). Macrophages play a crucial role in fibrosis, and macrophage receptors and ligands associated with fibrosis are being investigated as potential targets for anti-fibrotic drugs. Although current treatments for fibrotic diseases such as idiopathic fibrosis, systemic sclerosis, and musculoskeletal disease fibrosis typically target inflammatory responses, increasing evidence suggests that mechanisms driving fibrosis differ from those regulating inflammation (202).

Platelet-derived growth factor (PDGF) is a critical pro-fibrotic factor that binds to the PDGF receptor on the surface of macrophages, activating fibroblast proliferation and migration, thereby increasing ECM synthesis (203). Platelet-derived growth factors (PDGFs) occupy a central role in SSc-related fibrosis and represent potential molecular targets for systemic sclerosis (SSc) (199, 204). Receptor/ligand analysis of macrophage-mesenchymal progenitor cell (MPC) cross-talk reveals that under fibrotic conditions, transforming growth factor-β (TGF-β) is enhanced, while platelet-derived growth factor (PDGF) signaling is enhanced under regenerative conditions (101) providing targets for fibrosis treatment. Interleukin-13 (IL-13) is a Th2 cell factor that can activate the JAK-STAT pathway by acting on the IL-13 receptor on the surface of macrophages, promoting M2 macrophage polarization and leading to the development of pathological fibrosis (205, 206). IL-33 is a novel pro-fibrotic cytokine that signals through ST2, recruiting and directing inflammatory cell function and enhancing pro-fibrotic cytokine production through polarization of M2 macrophages in an ST2- and macrophage-dependent manner, generating IL-13 and TGF-β1, thereby promoting the onset and progression of fibrosis (102).

Peroxisome proliferator-activated receptors (PPARs) are important regulators of metabolism and inflammation (207, 208). PPARs can directly modulate macrophage activation without affecting infiltration, effectively counteracting inflammation and fibrosis progression (209). The pan-PPAR agonist lanifibranor has been shown to effectively improve tissue inflammation and fibrosis (103). C-C motif chemokine ligand 2 (CCL-2) binds to C-C motif chemokine receptor 2 (CCR2), promoting macrophage aggregation at damaged tissues. Macrophage infiltration exacerbates the inflammatory response, further promoting fibrosis development. Studies have shown that chemokine receptors CCR-2 and CX3CR1 regulate skin fibrosis in cytokine-induced systemic sclerosis mouse models, suggesting that blocking C-C motif chemokine ligand (CCL-24) or CCL-2 to inhibit monocyte recruitment may be an attractive new therapy to limit SSc fibrosis manifestations (104).

Transforming growth factor-β (TGF-β) is the most important cytokine in the pro-fibrotic process (210, 211). Macrophages express TGF-β receptors on their surface, which, upon binding to TGF-β, can activate macrophages and induce fibroblast transformation into a pro-fibrotic activated state. TGF-β signaling is considered a key pathway in almost all types of fibrosis (212). CCL-2/CCR-2 interactions also induce fibrosis *via* the TGF-β pathway (104). Since the approval of pirfenidone, targeting TGF-β signaling has been anticipated as an effective treatment for fibrosis (105, 212). The Wnt ligand Wnt3a enhances IL-4 or TGF-β1-induced M2 macrophage polarization. Wnt/β-catenin signaling works in conjunction with TGF-β signaling during fibrosis; TGF-β signaling can induce the expression of Wnt/β-catenin superfamily members and vice versa (106, 213). Drugs and small molecule inhibitors targeting the Wnt signaling pathway have demonstrated some efficacy in animal models of fibrotic diseases (106). Thus, inhibiting the Wnt signaling pathway may have therapeutic potential for anti-fibrotic treatment. These receptor-ligand interactions affecting fibrosis may become a suitable and promising therapeutic strategy in the future. As for the Wnt/β-catenin signaling pathway, some small molecule inhibitors have been developed, among which MSAB is an effective and selective inhibitor of Wnt/β-catenin signal transduction. MSAB binds with β-catenin, promoting its degradation and specifically downregulating Wnt/β-catenin target genes (214). TGFβ1-IN-1 (Compound 42) is an effective orally active inhibitor of TGF-β1. TGFβ1-IN-1 inhibits the upregulation of fibrosis markers (α-SMA and fibronectin) induced by TGF-β1, making it suitable for fibrotic disease research. *In vivo* studies have shown that TGFβ1-IN-1 inhibits TGF-β1-induced tissue damage and fibrosis, suppresses the activation of epithelial-mesenchymal transition (EMT), and improves the immune microenvironment of tissues (215). In clinical settings, the effects of these inhibitors vary across different diseases and conditions. Some early studies indicate that inhibitors of the Wnt/β-catenin and TGF-β signaling pathways, such as SSc, have shown some efficacy in certain musculoskeletal diseases (215). However, there have yet to be large-scale, randomized controlled trials to verify the effectiveness and safety of these drugs in a clinical setting.

## 4 Targeted macrophage therapeutic strategies

Chimeric antigen receptor T (CAR-T) cell therapy has achieved evolutionary success in hematologic malignancies and has expanded its application to solid tumors (216). However, adoptive T-cell therapy (ACT) for autoimmune diseases requires a high degree of specificity to avoid attacking healthy tissue and identifying and targeting pathogenic T-cell subpopulations remains challenging (217). Furthermore, in musculoskeletal diseases, the therapeutic goal is to restore immune system balance rather than simply enhancing or suppressing immune responses.

**Figure 3:**
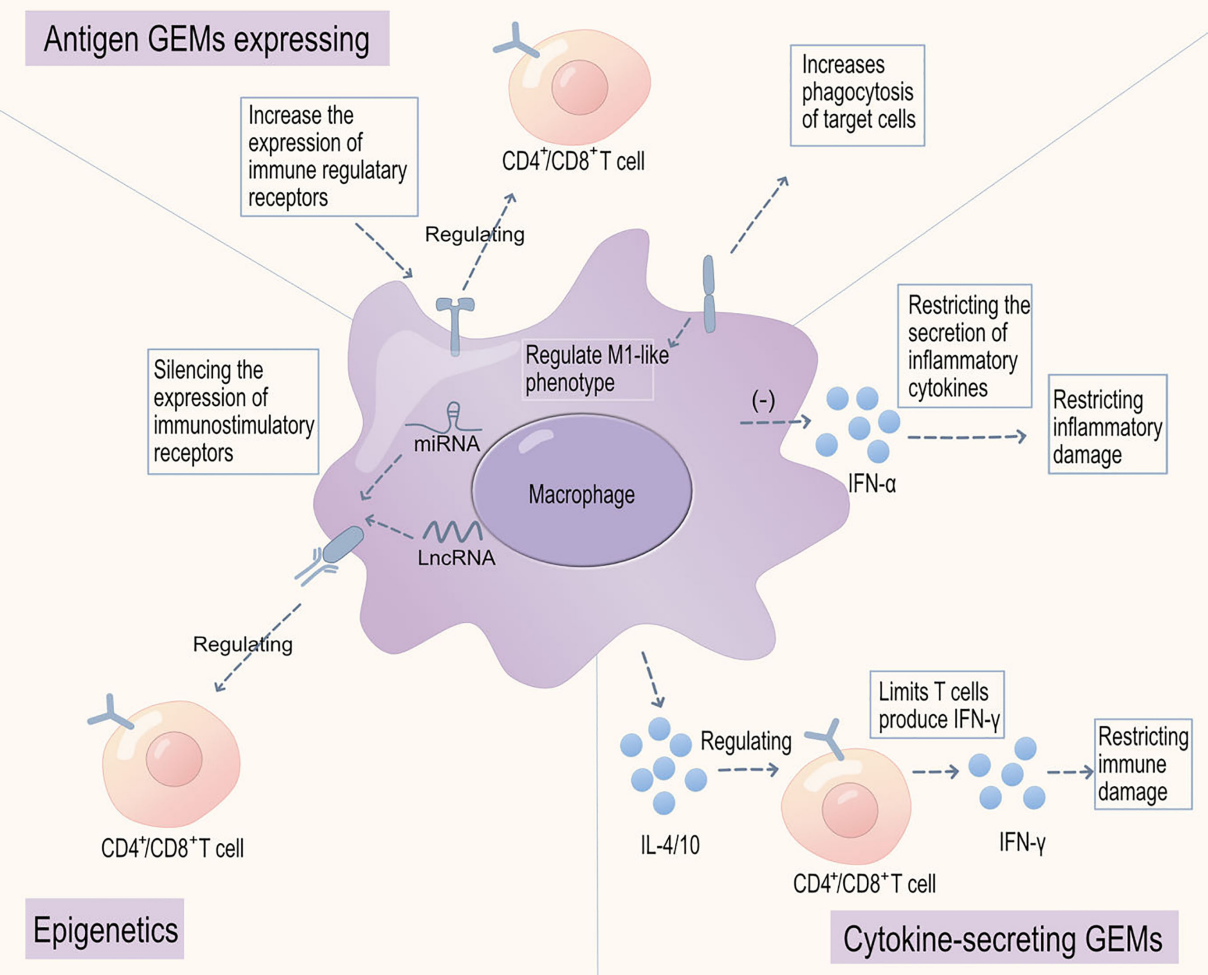
Genetically engineered macrophages for the treatment of musculoskeletal disorders. There are three major categories of genetically engineered macrophages (GEMs): firstly, those with engineered cell surface molecules, including increased expression of membrane regulatory receptors and MOTO-CAR with TGF-βR or IL-10R receptor signaling domains, to modulate immune cell activation, reduce immune-inflammatory injury, or enhance macrophage phagocytic checkpoints for apoptotic cell clearance, and reduce autoantigen presentation and immune-inflammatory injury; secondly, those engineered to secrete inflammatory mediators and cytokines, such as increasing the secretion of immunoregulatory cytokines IL-4/10 to enhance immune cell regulation and limit interferon-α (IFNα) secretion, restricting immune-inflammatory injury; finally, those that can silence the expression of immune-stimulating receptors through epigenetics, alleviating the stimulatory effect on activated immune cells and reducing immune-inflammatory injury.

We have summarized the key approaches for utilizing genetically engineered macrophages in the treatment of musculoskeletal disorders (**Figure 3**). Genetically engineered macrophages (GEMs) may minimize the impact on the immune microenvironment and improve both innate and adaptive immune responses, making them suitable for treating musculoskeletal diseases such as rheumatoid arthritis and systemic lupus erythematosus (218, 219). Lentiviral expression systems have been validated for generating transduced monocytes and monocyte-derived macrophages, and transgene expression has been demonstrated to be stable over several weeks to months *in vitro* and in mouse xenograft models of GBM (220). GEMs employ the clustered regularly interspaced short palindromic repeats (CRISPR)/Cas9 system for gene editing to correct gene mutations and improve disease, such as by expressing TGF-βR or IL-10R to modulate immune cell activation (221–223). Modulation of immune responses is also possible by using the CRISPR system to knock out genes that cause aberrant activation of cytotoxic cell functions, including IL-10R and PD-1R (224, 225). These results suggest that GEMs are an ideal approach for manipulating the immune microenvironment and suppressing immune damage. CARs may potentially contribute to the treatment of some common musculoskeletal genetic mutation diseases, such as cystic fibrosis and amyotrophic lateral sclerosis.

Furthermore, the use of epigenetic RNA interference (such as miRNA or lncRNA) can specifically reduce the expression of particular receptors (226). This approach is achieved by degrading the target gene’s miRNA, thereby affecting receptor protein synthesis. The exploration and pathway analysis of microRNAs (miRNAs) have paved the way for discovering potential therapeutic targets (226, 227). miRNAs are small non-coding oligonucleotides characterized by their role in gene regulation, transcription, and immune modulation mechanisms (228). Research on lncRNAs has underscored their importance as both immune markers of active disease progression and immune modulators of innate processes such as apoptosis and autophagy (229). Epigenetic silencing of miRNAs remodels macrophages through receptor expression or paracrine secretion of cytokines, such as macrophage migration inhibitory factor (230).

In addition to engineering macrophages to express cell surface receptors, research has also focused on modulating the expression of ligand cytokines that regulate the immune microenvironment and immune cell activation (231, 232). Genetically engineered macrophages secreting anti-inflammatory cytokines, such as interleukin-4 (IL-4) and interleukin-10 (IL-10), effectively reach the primary site, significantly alleviate inflammatory responses, and protect tissue cells from LPS-induced functional impairment, suggesting that these engineered macrophages may have inhibitory effects on inflammatory damage (233, 234). Furthermore, in preclinical models, macrophages carrying engineered particles containing interferon-γ (referred to as “backpacks”) exhibit enhanced phagocytic activity in the immune microenvironment, polarizing macrophages towards a pro-inflammatory phenotype, resulting in sustained phagocytic function enhancement and reduced self-antigen presentation (235, 236).

Concerning these strategies, future research may continue to explore how to apply these methods in clinical settings, such as using macrophage-based therapies in musculoskeletal diseases or other inflammatory diseases. In addition, further research is needed on how to precisely manipulate the polarization and function of macrophages *in vivo*, such as by developing new drugs or cell therapies. Overall, the field of genetically engineered macrophages has opened an exciting avenue for targeting the immune damage microenvironment in musculoskeletal diseases. However, the heterogeneity and plasticity of macrophage subpopulations need to be carefully analyzed in clinical and preclinical models to optimize specific clinical responses.

## 5 Conclusion and perspectives

In this review, we have focused on the various functions of macrophage receptor-ligand interactions, which serve as potential targets for immune-related musculoskeletal disease therapies. However, the development of such therapies is challenged by increasing complexity on several levels.

First, all these receptor-ligand interactions are intricately interconnected. On the one hand, macrophage receptors, upon stimulation by ligands such as cytokines or other molecules, activate or inhibit a series of signaling pathways, thereby influencing macrophage polarization and function. On the other hand, the polarization state and function of macrophages can also affect the expression of their surface receptors, which in turn further influences receptor-ligand interactions (237).

For instance, receptor-ligand interactions regulate multiple aspects of macrophage polarization and function, including aggregation, phagocytosis, and macrophage-derived products that, in turn, regulate receptor-ligand interactions (51, 73). A second, largely unexplored area is the determination of specific macrophage phenotypes in particular etiologies or pathological processes by selectively altering macrophage phenotype and function (29, 70). Lastly, the persistence of macrophage-directed therapy effects is a prominent challenge that will impact whether macrophage-targeted therapies can serve as standalone treatments or only in conjunction with other forms of therapeutic targets (238). Crosstalk among these receptors in the immune microenvironment must be considered to enhance efficacy and minimize off-target toxicological effects. Additionally, due to the marked diversity and expression of human and murine myeloid cell subpopulations, the relevance of inhibiting receptors in human myeloid cell subpopulations requires careful evaluation.

Macrophage-targeted therapy has several undeniable advantages in the treatment of musculoskeletal diseases: first, macrophages can suppress excessive immune responses and restore immune system balance in the treatment of musculoskeletal diseases through the release of anti-inflammatory cytokines and promotion of regulatory T-cell proliferation, among other pathways (44, 239). Second, macrophages exhibit high plasticity and can differentiate into subtypes with distinct functions and phenotypes in response to environmental signals and stimuli. By modulating macrophages ex vivo, they can be directed to differentiate into immune-regulatory phenotypes, ultimately improving musculoskeletal disease conditions (8). Lastly, macrophage therapy allows for personalized treatment plans for each patient. Ex vivo modulation and genetic engineering of macrophages can provide patient-specific treatment strategies to address different types of musculoskeletal diseases, enhancing specificity for disease treatment while minimizing the impact on healthy tissues (240, 241).

In conclusion, the field of immune therapy for musculoskeletal diseases has already provided benefits to many patients, and these studies will enable the next wave of musculoskeletal disease immunotherapies targeting macrophage subpopulations to further enhance immune responses and clinical outcomes. The beneficial effects of adoptive polarized macrophage transfer therapy are currently being evaluated, with significant improvements observed in several different animal models. Another promising strategy is the treatment of autoimmune diseases with miRNA-based epigenetic therapies. The high plasticity of macrophages allows them to alter their effector functions, and therefore, they can potentially be manipulated to inhibit chronic inflammatory immune damage and fibrotic processes.

## Author contributions

JZ wrote the initial manuscript. CF and JZ contributed new ideas. JF and HW created the figures. JF and ZF created **Table 1**. YX, YL, ZF, and HW revised the manuscript. All authors contributed to the article and approved the submitted version.
